# Supplementary material for: The Red Sea Deep Water is a potent source of atmospheric ethane and propane
Source: Nat Commun. 2020 Jan 28;11:447. doi: 10.1038/s41467-020-14375-0 (PMC6987153; doi:10.1038/s41467-020-14375-0)
Supplement: Supplementary file 1 — Supplementary Information [file 41467_2020_14375_MOESM1_ESM.pdf]

Supplementary information for

THE RED SEA DEEP WATER IS A POTENT SOURCE OF ATMOSPHERIC ETHANE AND PROPANE

by BOURTSOUKIDIS et al.

Supplementary Figures

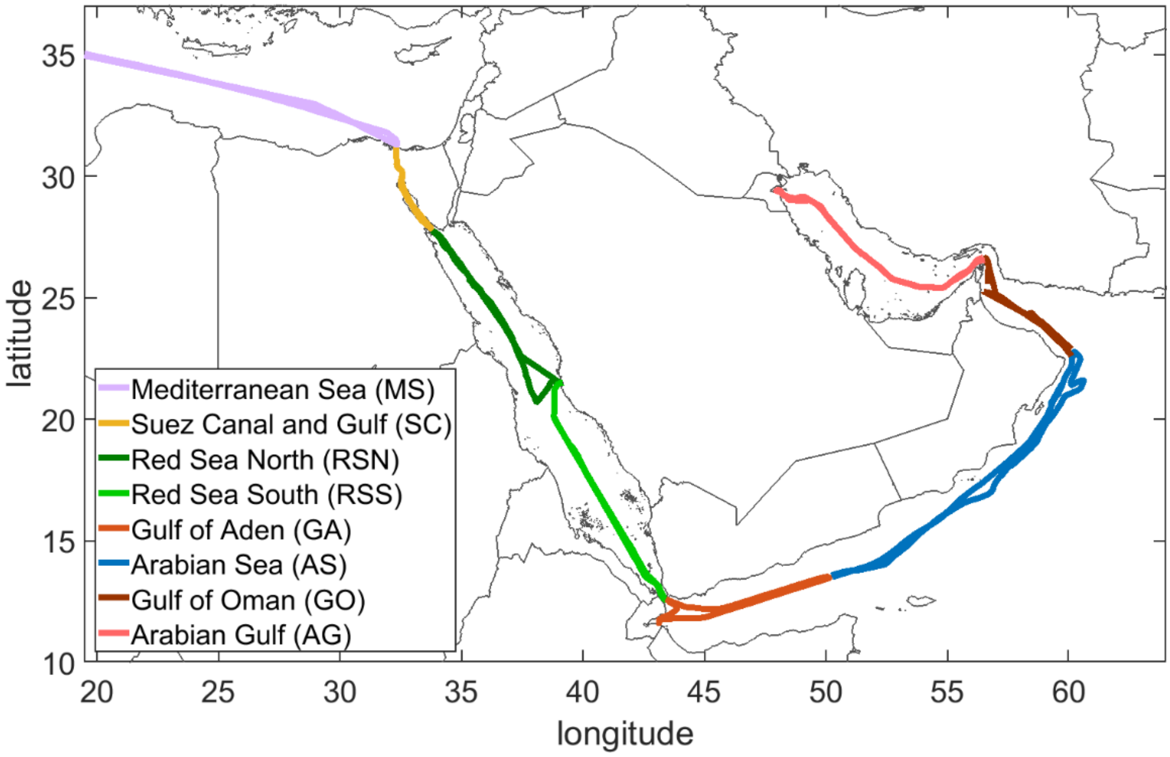

Supplementary Figure 1. Geographical demarcation along the route for both legs of the campaign. Mediterranean Sea (MS; from 35°57'32.4"N 15°00'46.8"E to 31.256 N, 32.354 E), Suez Canal and Gulf (SC; from 31°15'21.6"N 32°21'14.4"E to 27°49'15.6"N 33°45'57.6"E), Northern Red Sea (RSN; from 27°49'15.6"N 33°45'57.6"E to 21°25'33.6"N 38°58'19.2"E), Southern Red Sea (RSS; from 21°25'33.6"N 38°58'19.2"E to 12°33'10.8"N 43°24'46.8"E), Gulf of Aden (GA; from 12°33'10.8"N 43°24'46.8"E to 13°27'39.6"N 50°08'31.2"E), Arabian Sea (AS; from 13°27'39.6"N 50°08'31.2"E to 22°52'08.4"N 60°06'18.0"E), Gulf of Oman (GO; from 22°52'08.4"N 60°06'18.0"E to 26°36'57.6"N 56°33'57.6"E), and Arabian Gulf (AG; from 26°36'57.6"N 56°33'57.6"E to 29°22'51.6"N 47°57'10.8"E).

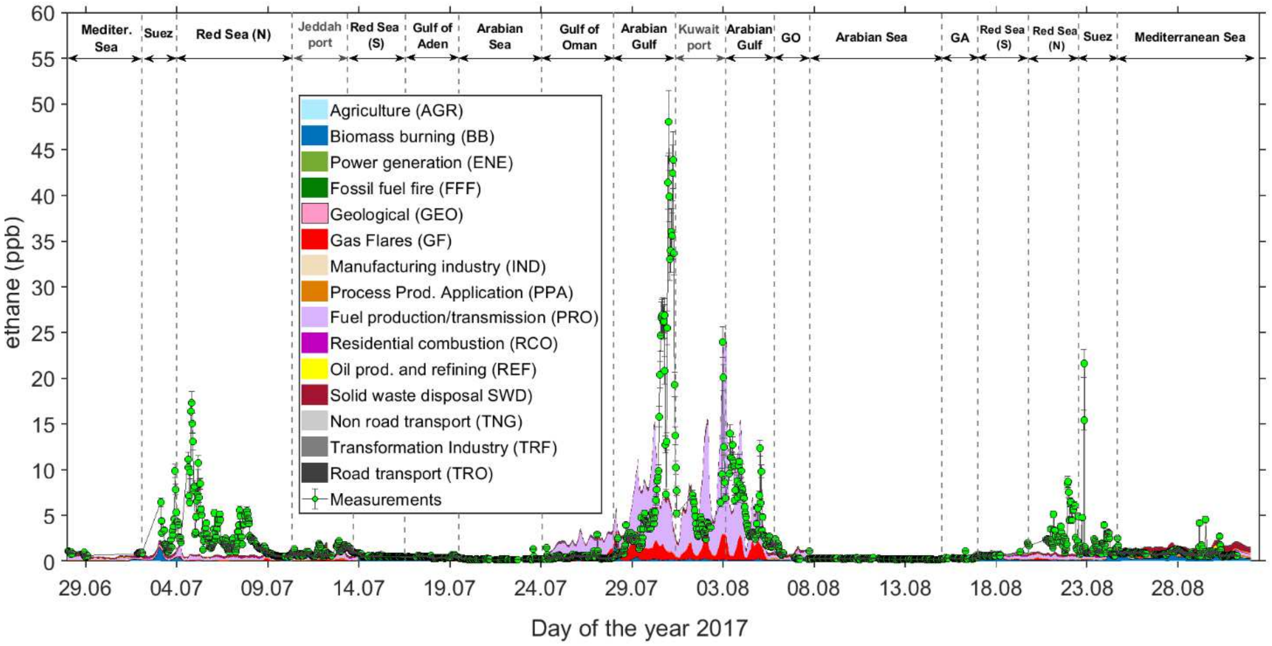

Supplementary Figure 2. Timeline of measured (green circles) and simulated (colored areas) ethane mixing ratios along the route. The error bars indicate the uncertainty of each measured sample.

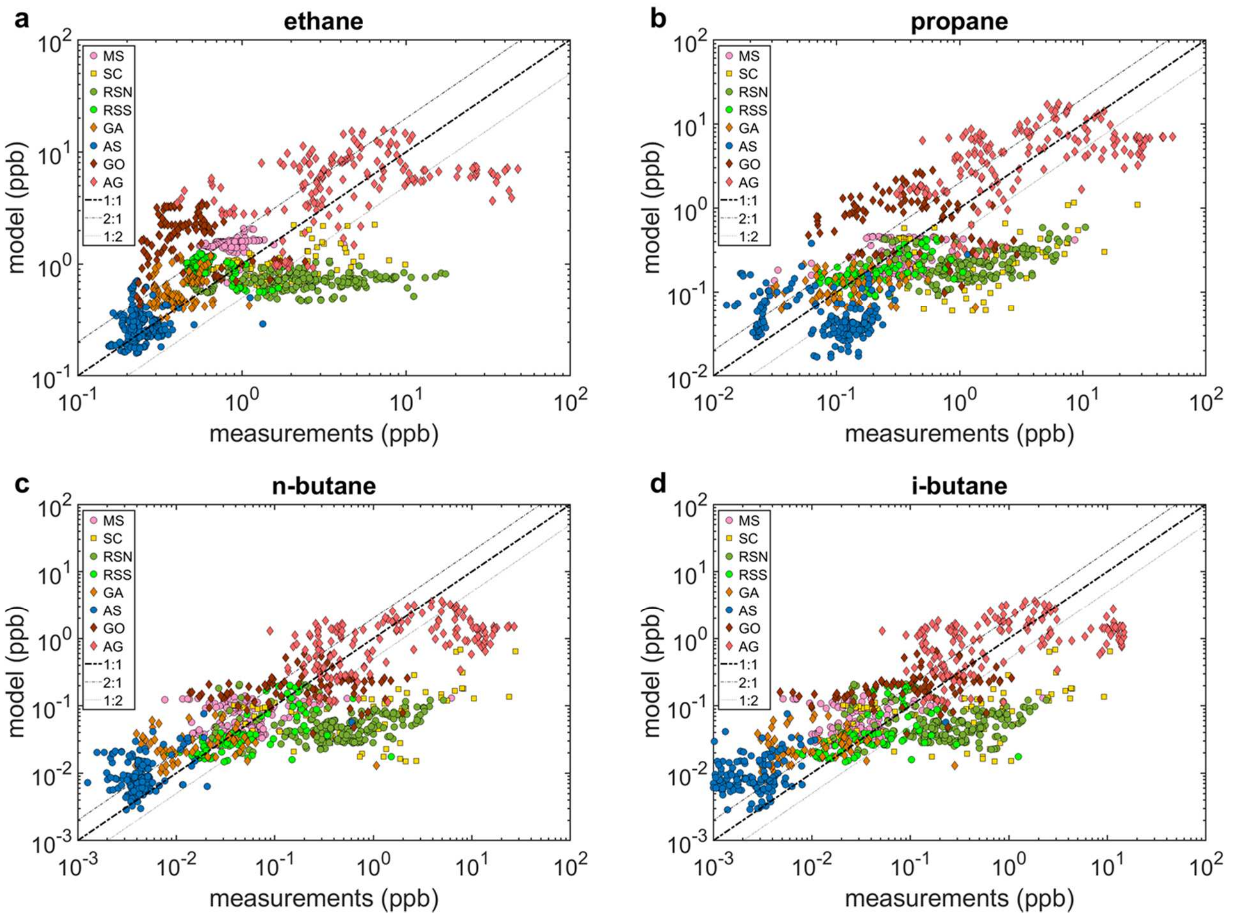

Supplementary Figure 3. Intercomparison between measured and simulated volume mixing ratios for ethane (**a**), propane (**b**), n-butane (**c**) and i-butane (**d**). The abbreviations indicate the respective regions. MS: Mediterranean Sea, SC: Suez Canal, RSN: Red Sea North, RSS: Red Sea South, GA: Gulf of Aden, AS: Arabian Sea, GO: Gulf of Oman, and AG: Arabian Gulf.

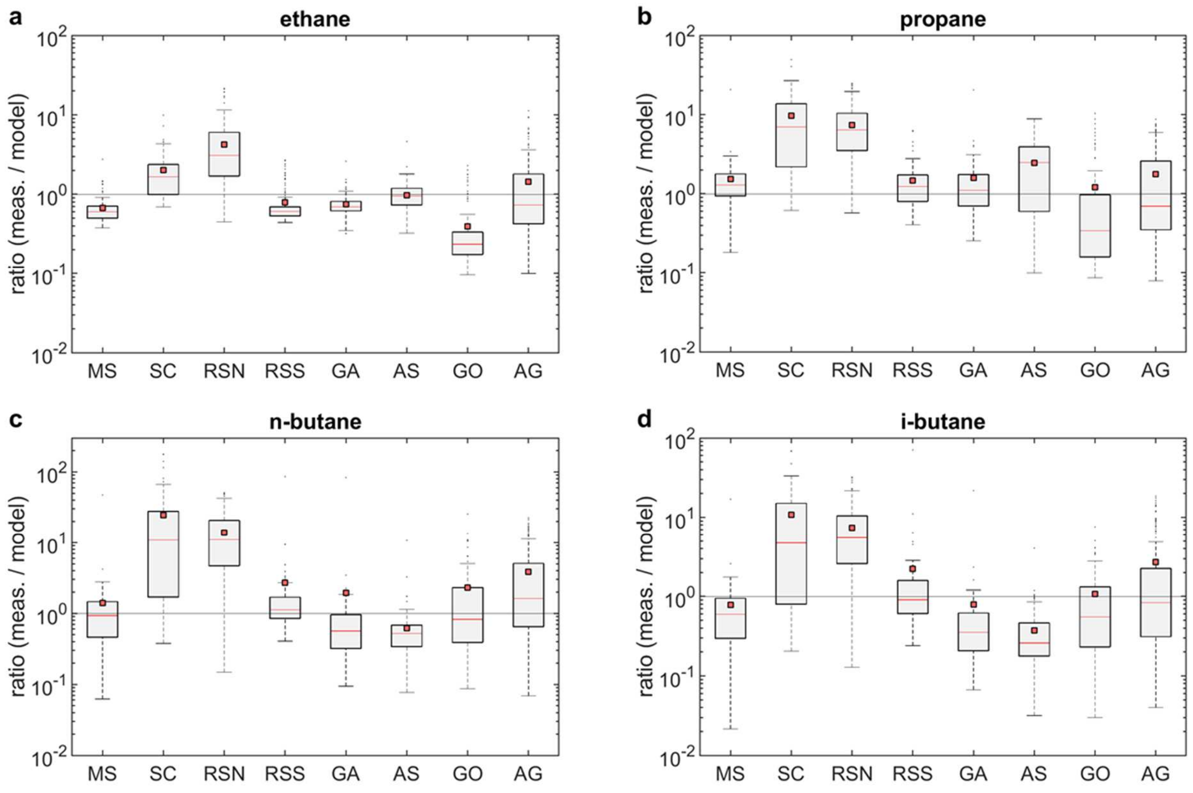

Supplementary Figure 4. Boxplot statistics between measured and simulated volume mixing ratios for ethane (**a**), propane (**b**), n-butane (**c**) and i-butane (**d**) for each region (MS: Mediterranean Sea, SC: Suez Canal, RSN: Red Sea North, RSS: Red Sea South, GA: Gulf of Aden, AS: Arabian Sea, GO: Gulf of Oman, and AG: Arabian Gulf). Regional ratio statistics are displayed with the boxplots that illustrate the median with red line and the mean with red squares. The bottom and top edges of the box indicate the 25th (q1) and 75th (q3) percentiles, respectively. The boxplot draws points as outliers if they are greater than q3+w×(q3–q1) or less than q1–w×(q3–q1). The whiskers (w) correspond to ±2.7σ and 99.3 % coverage if the data are normally distributed.

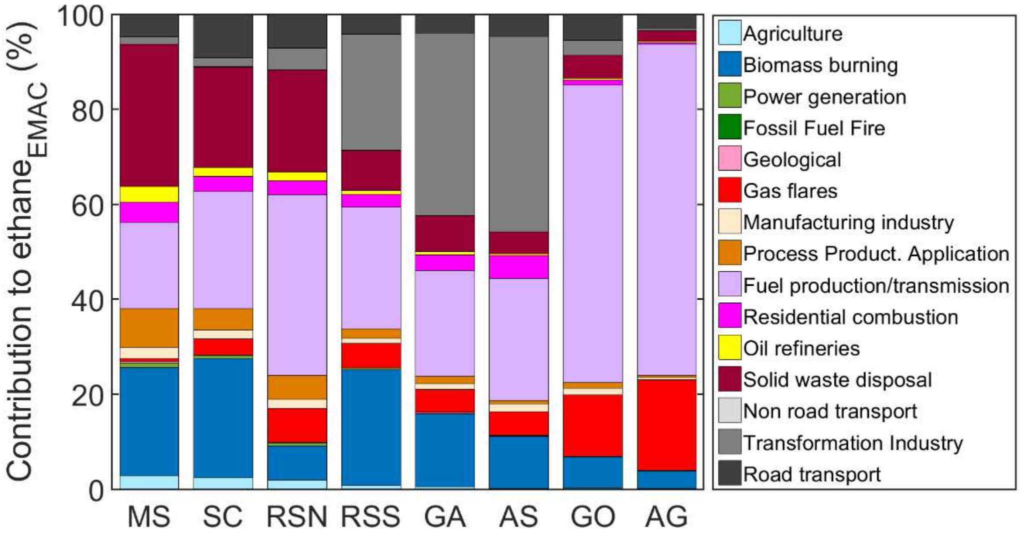

Supplementary Figure 5. Normalized source contribution for model simulations for all regions. MS: Mediterranean Sea, SC: Suez Canal, RSN: Red Sea North, RSS: Red Sea South, GA: Gulf of Aden, AS: Arabian Sea, GO: Gulf of Oman, and AG: Arabian Gulf.

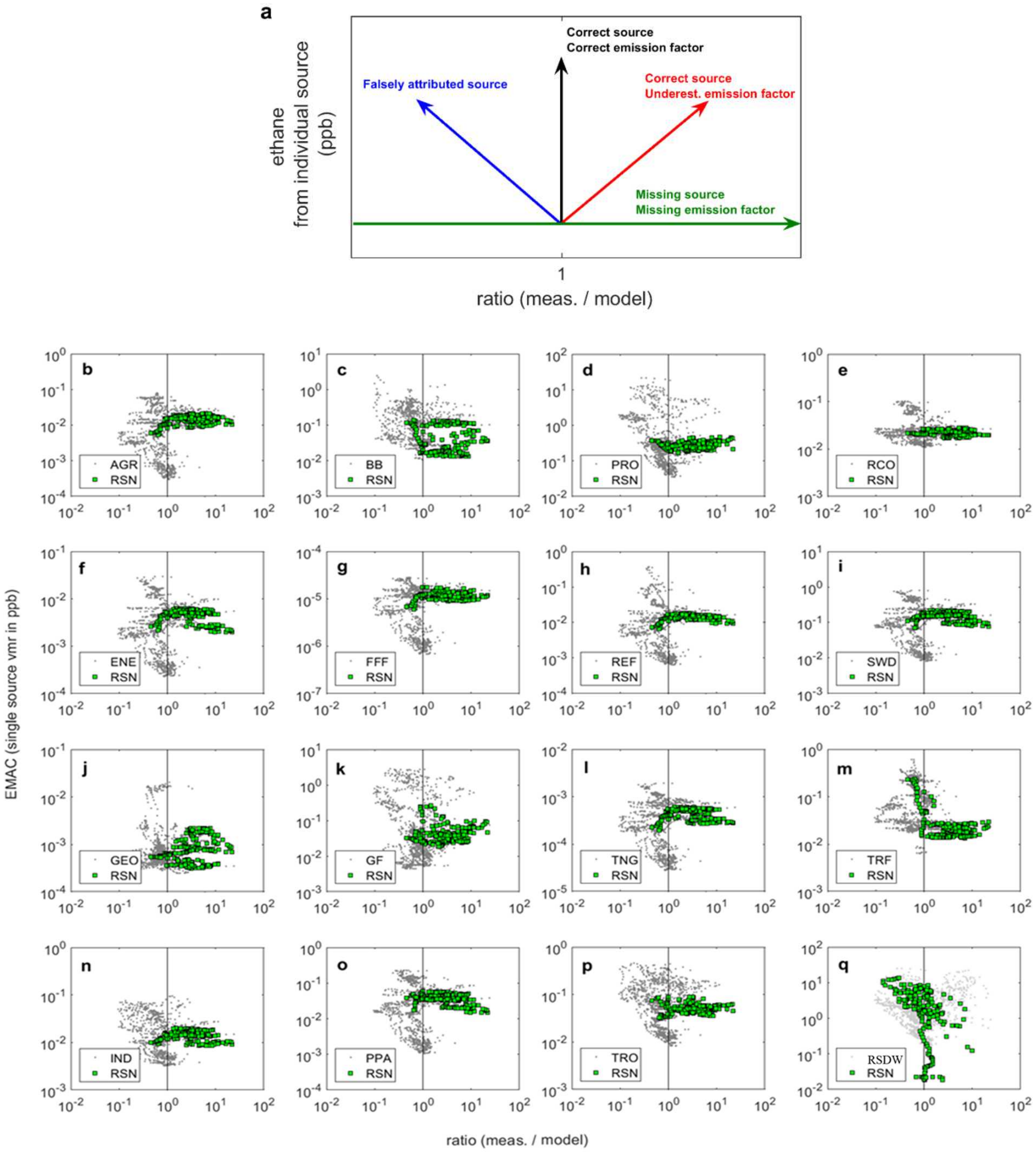

Supplementary Figure 6. Single source investigations. **a** Conceptual trends of data point evolution as a function of the measurements to simulated ratios and EMAC single source volume mixing ratio within the model. The relationship is presented for all existing sources in EMAC inventories (**b** agriculture (AGR), **c** biomass burning (BB), **d** fuel production and transmission (PRO), **e** residential combustion (RCO), **f** power generation (ENE), **g** fossil fuel fire (FFF), **h** oil refineries (REF), **i** solid waste disposal (SWD), **j** geological (GEO), **k** gas flares (GF), **l** non road transport (TNG), **m** transformation industry (TRF), **n** manufacturing industry (IND), **o** process emissions during production and application (PPA), **p** road transport (TRO)) as well as for the Red Sea Deep Water (RSDW) (**q**) that were included after the quantification of their emission rates (see Fig. 3).

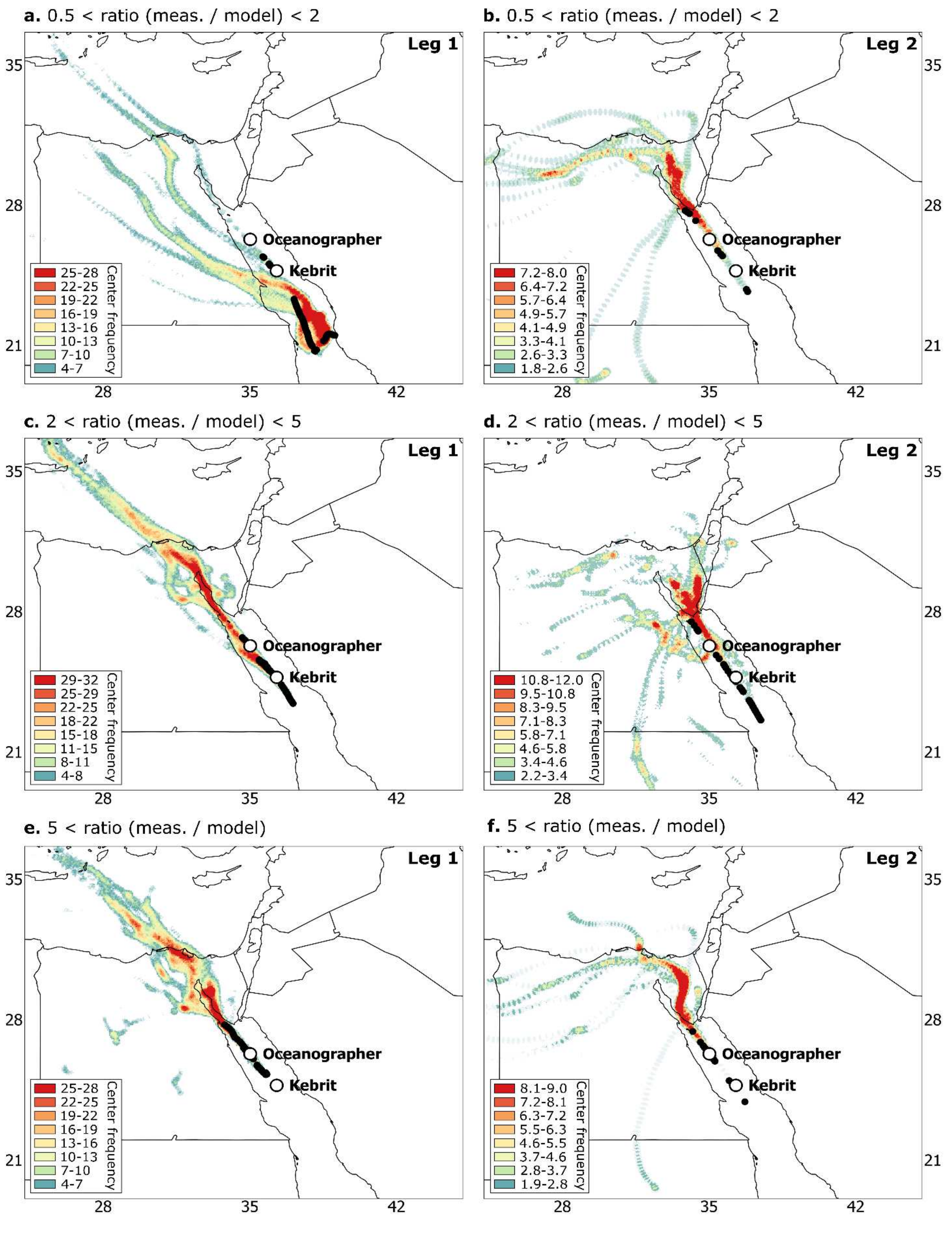

Supplementary Figure 7. Back-trajectory density plots for the ratio between measured and simulated ethane. Clustered data points illustrate the 3-day back-trajectories (calculated using HYSPLIT[16]) for model discrepancy by a factor of 2 (**a** and **b**), for ratios between 2 and 5 (**c** and **d**), and for ratios higher than 5 (**e** and **f**) for leg 1 (left) and leg 2 (right).

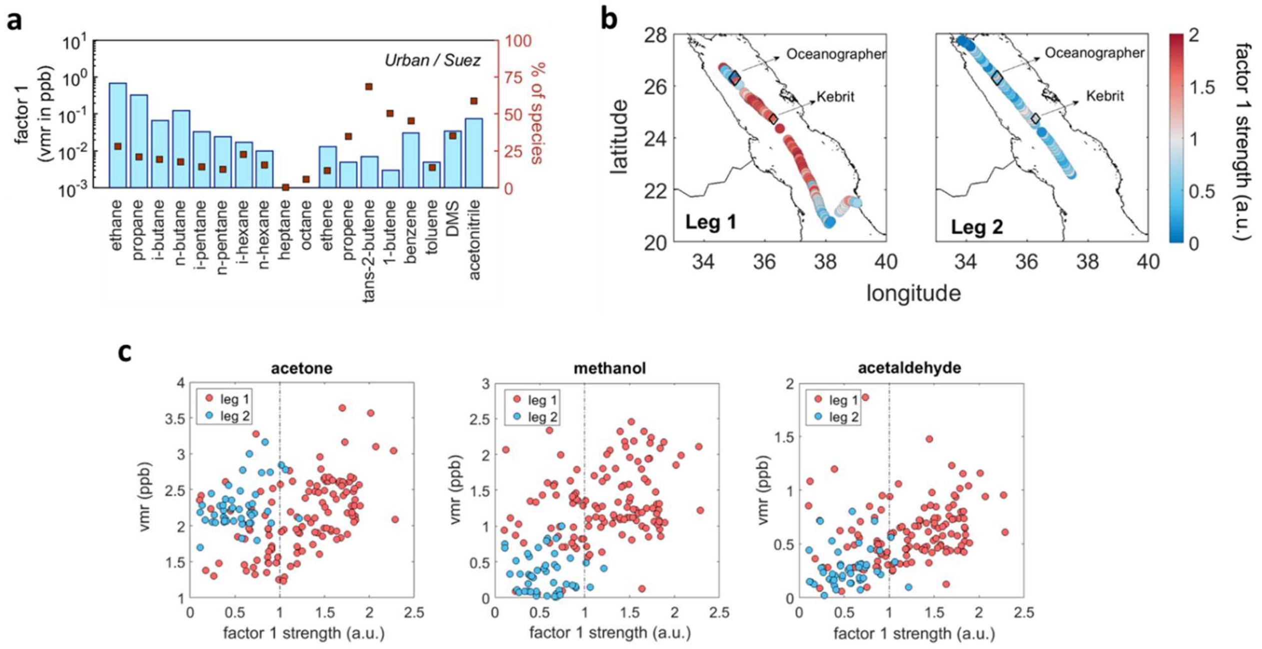

Supplementary Figure 8. Source apportionment for the northern Red Sea region. **a** Profile of factor 1 (Urban / Suez) derived from Positive Matrix Factorization (PMF) analysis. The blue bars indicate the volume mixing ratio contribution from each source and the brown squares the % contribution of each species to the respective factor (sum = 100 %). In **b**, the factor 1 strength timelines are illustrated for both legs. In **c**, the strength of factor 1 (average strength = 1) is correlated with acetone, methanol and acetaldehyde for leg 1 (red circles) and leg 2 (blue circles).

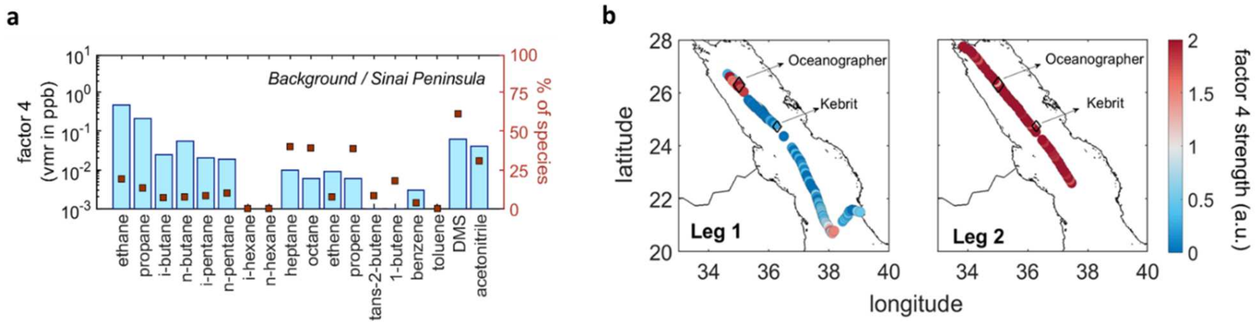

Supplementary Figure 9. Source apportionment for the northern Red Sea region. **a** Profile of factor 4 (Background / Sinai Peninsula) derived from Positive Matrix Factorization (PMF) analysis. The blue bars indicate the volume mixing ratio contribution from each source and the brown squares the % contribution of each species to the respective factor (sum = 100 %). In **b**, the factor 4 strength (average strength = 1) timelines are illustrated for both legs.

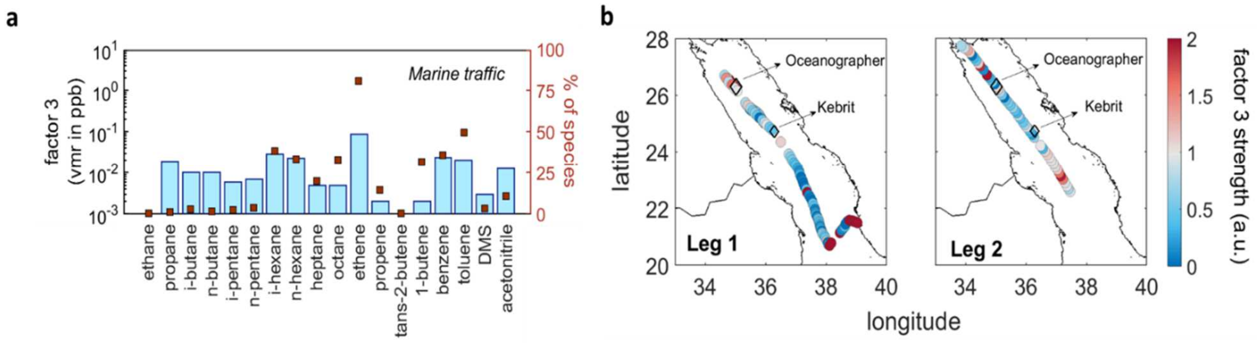

Supplementary Figure 10. Source apportionment for the northern Red Sea region. **a** Profile of factor 3 (Marine traffic) derived from Positive Matrix Factorization (PMF) analysis. The blue bars indicate the volume mixing ratio contribution from each source and the brown squares the % contribution of each species to the respective factor (sum = 100 %). In **b**, the factor 3 strength (average strength = 1) timelines are illustrated for both legs.

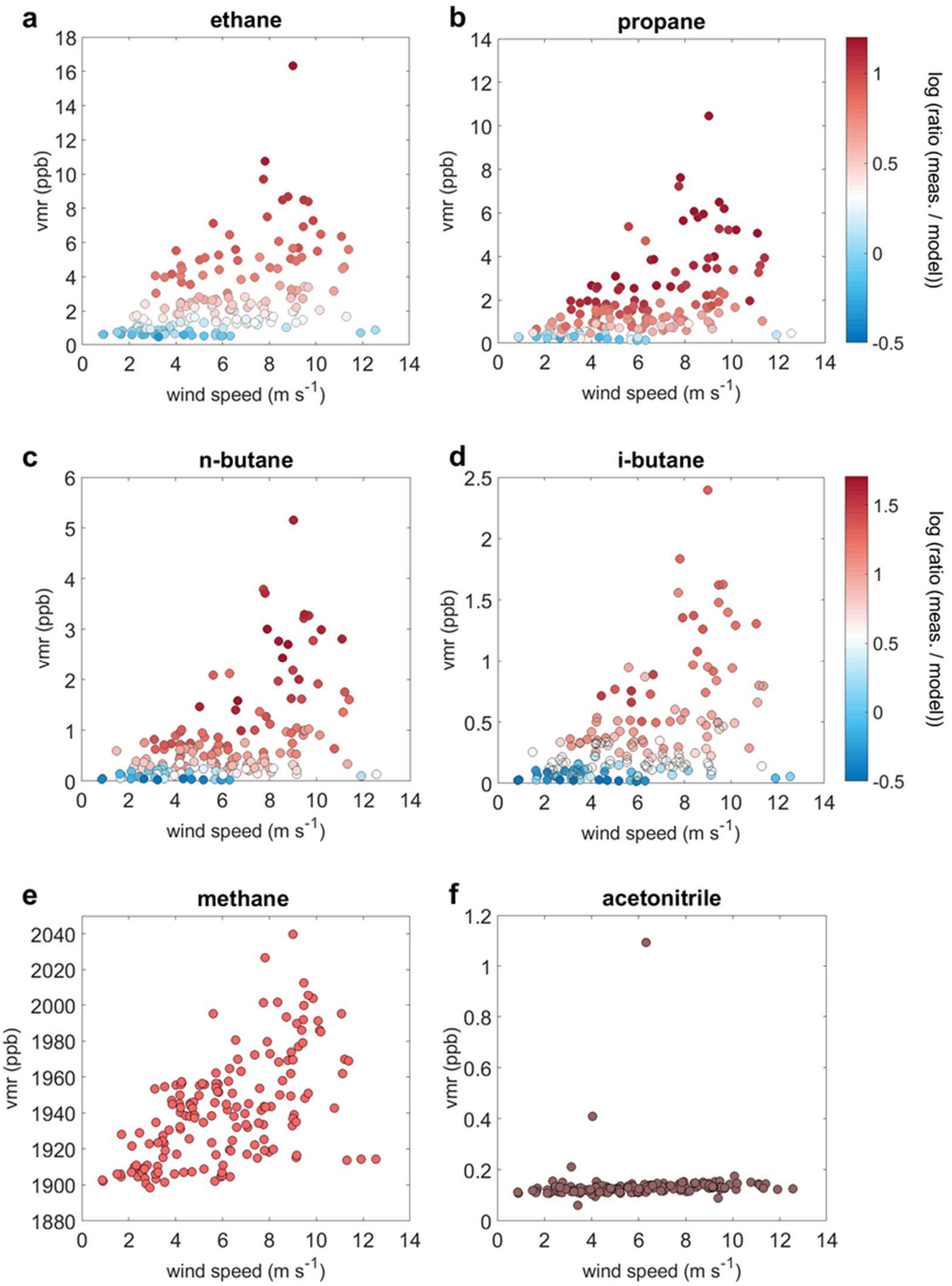

Supplementary Figure 11. Relationship between wind speed and measured volume mixing ratios of ethane (**a**), propane (**b**), n-butane (**c**), i-butane (**d**), methane (**e**) and acetonitrile (**f**).

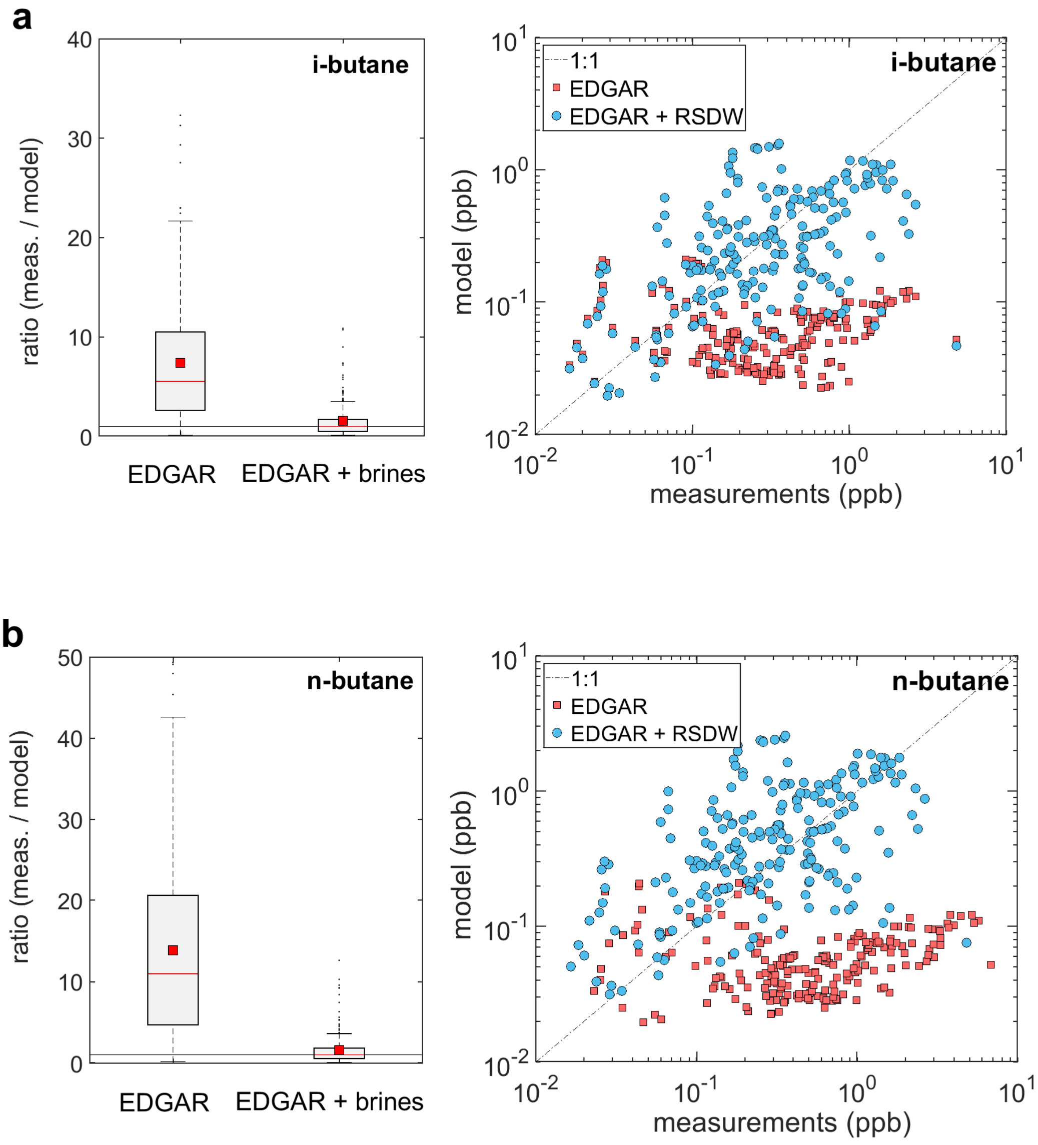

Supplementary Figure 12. Comparison between model simulations and measurements over the northern Red Sea region. Ethane (**a**) and propane (**b**) relationships are illustrated for the EDGAR v4.3.2 inventory and compared with the addition of the Red Sea Deep Water (RSDW) emissions in the inventory input. The boxplots that illustrate the median with red lines and the mean with green squares. The bottom and top edges of the box indicate the 25th (q1) and 75th (q3) percentiles, respectively. The boxplot draws points as outliers if they are greater than q3+w×(q3–q1) or less than q1–w×(q3–q1). The whiskers correspond to ±2.7σ and 99.3 % coverage if the data are normally distributed. The horizontal line indicates the 1:1 measurements to model ratio.

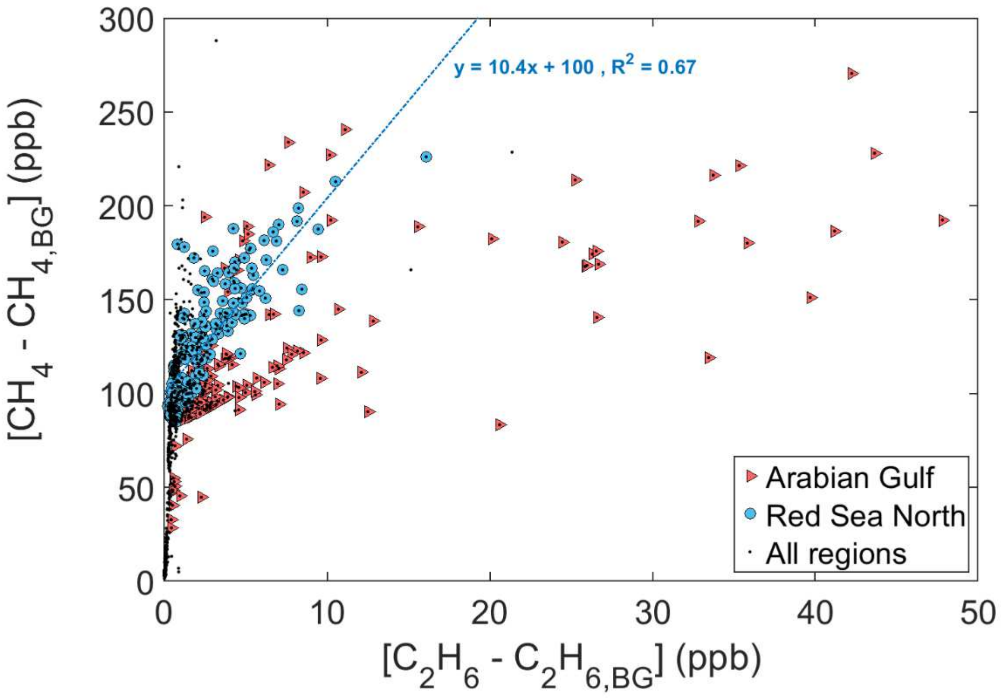

Supplementary Figure 13. Relationship between ethane and methane enhancement mixing ratios (i.e. subtracted regional background (BG)).

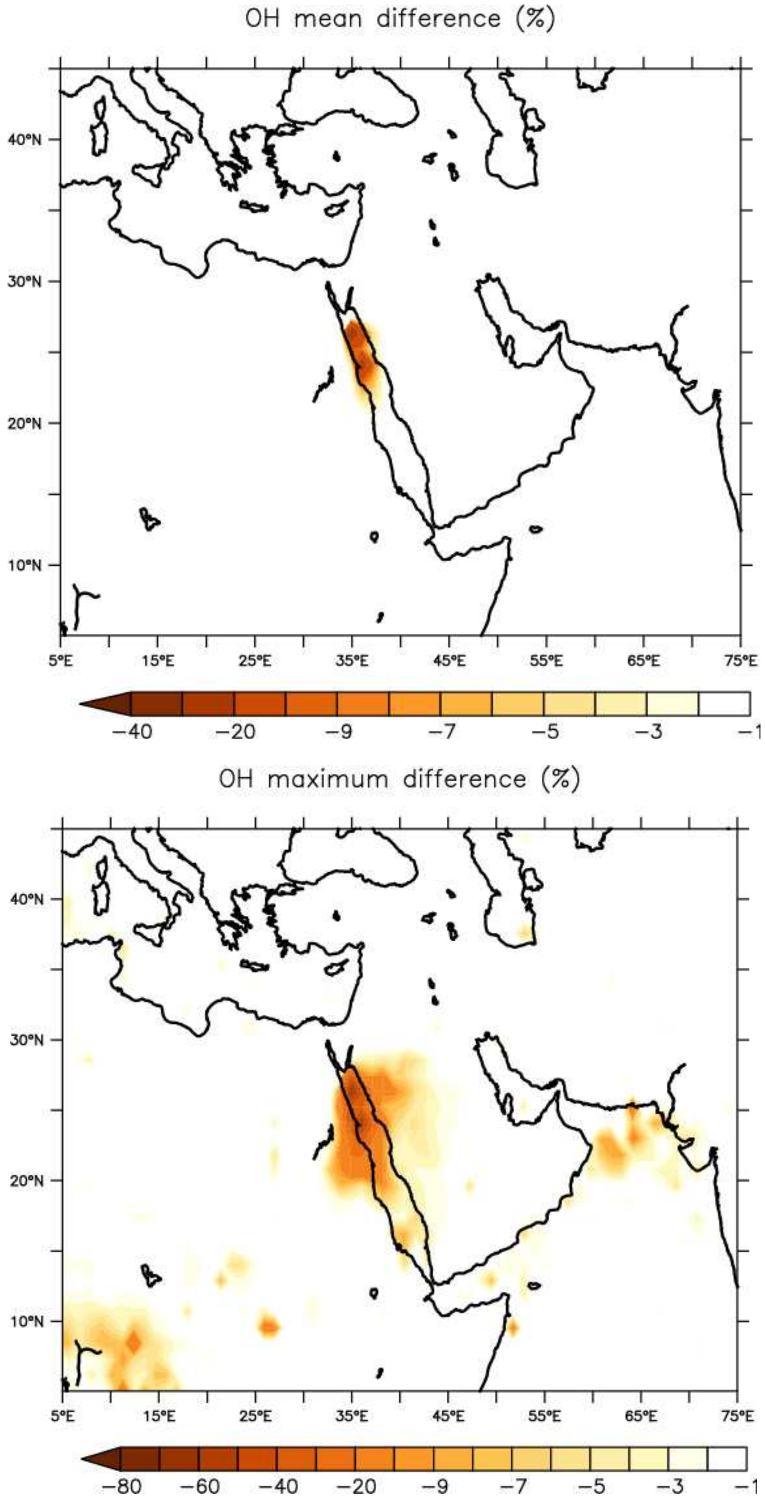

Supplementary Figure 14. Implications of Red Sea Deep Water (RSDW) emissions on summertime (June, July, and August) OH abundance. The mean fractional deviations (%) illustrates the spatial average differences (resolution of 1 hour) over the three-month simulations (June, July, August) using the model with and without the RSDW sources. The minimum differences illustrate the most relatively largest hourly differences between the two model simulations (original EDGAR v.4.3.2 minus the revised emission inventory).

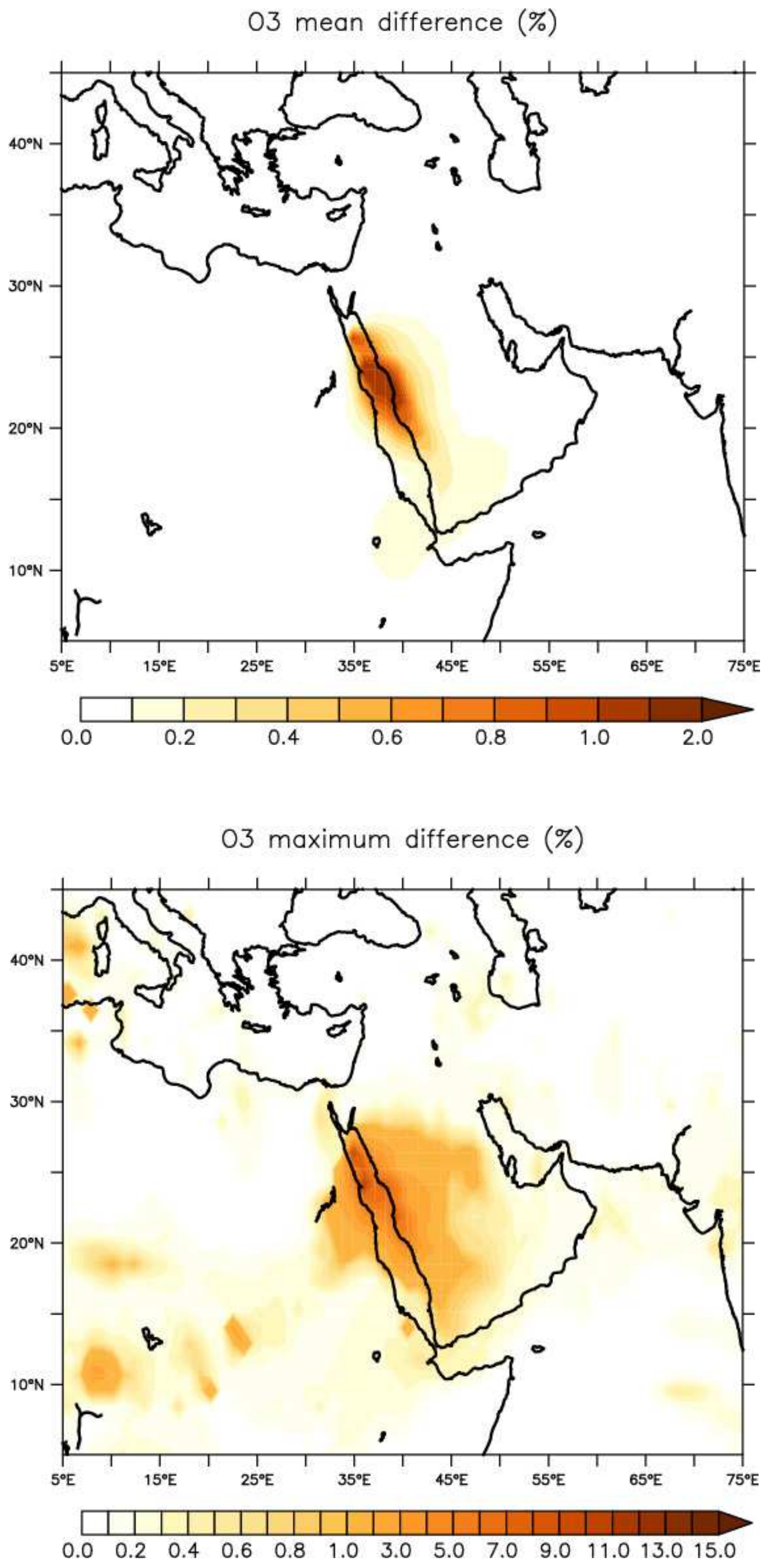

Supplementary Figure 15. Implications of Red Sea Deep Water (RSDW) emissions on summertime (June, July, and August) O_3 abundance. The mean fractional deviations (%) illustrates the spatial average differences (resolution of 1 hour) over the three-month simulations (June, July, August) using the model with and without the RSDW sources. The maximum differences illustrate the most relatively largest hourly differences between the two model simulations (original EDGAR v.4.3.2 minus the revised emission inventory).

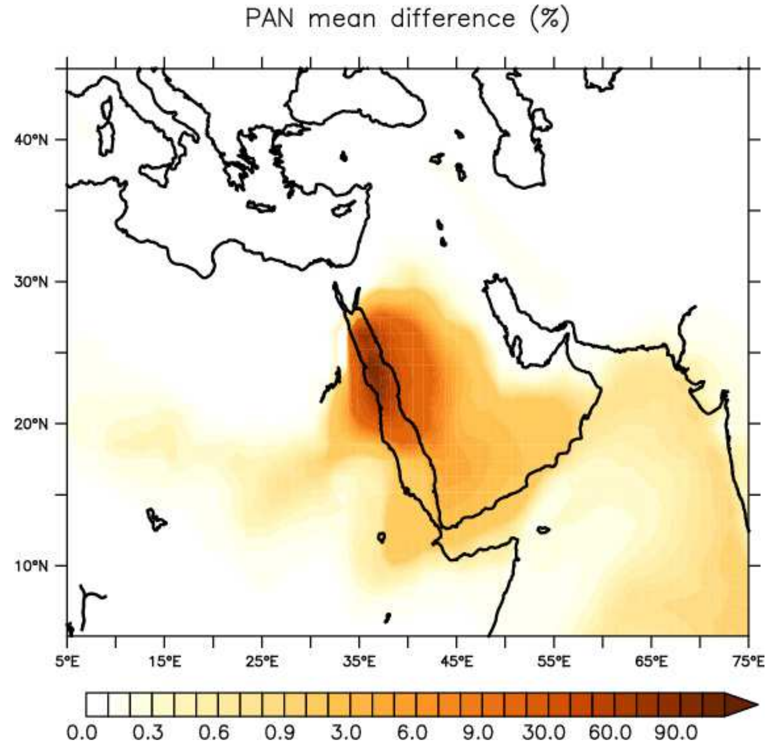

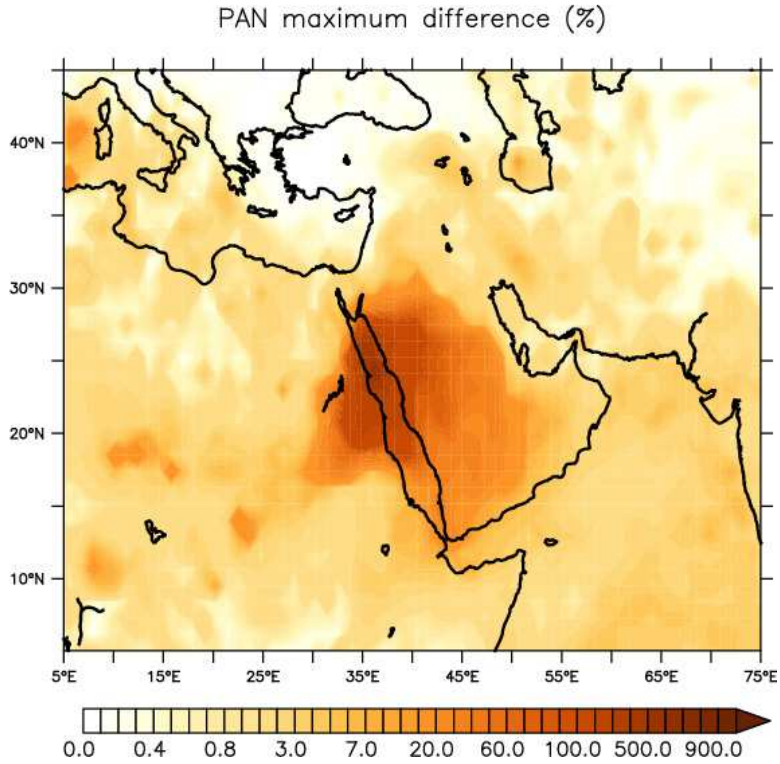

Supplementary Figure 16. Implications of Red Sea Deep Water (RSDW) emissions on summertime (June, July, and August) PAN abundance. The mean fractional deviations (%) illustrates the spatial average differences (resolution of 1 hour) over the three-month simulations (June, July, August) using the model with and without the RSDW sources. The maximum differences illustrate the most relatively largest hourly differences between the two model simulations (original EDGAR v.4.3.2 minus the revised emission inventory).

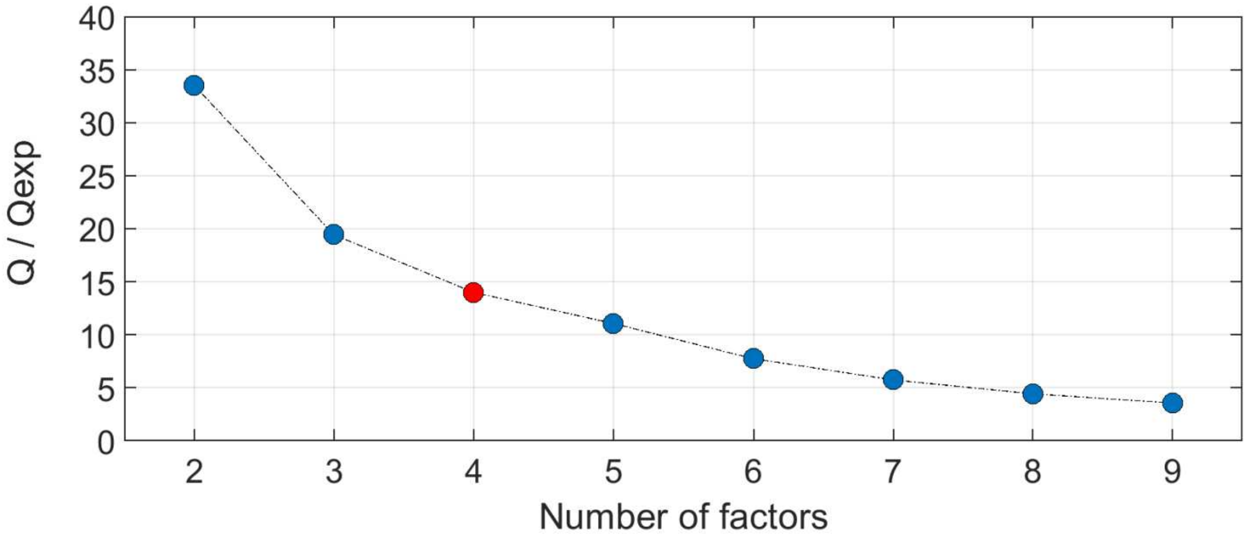

Supplementary Figure 17. PMF diagnostic Q/Q expected plot. Q = the sum of squared scaled residuals over the whole dataset, plotted versus the number of factors used in the PMF solution. Red circle indicates the optimum solution.

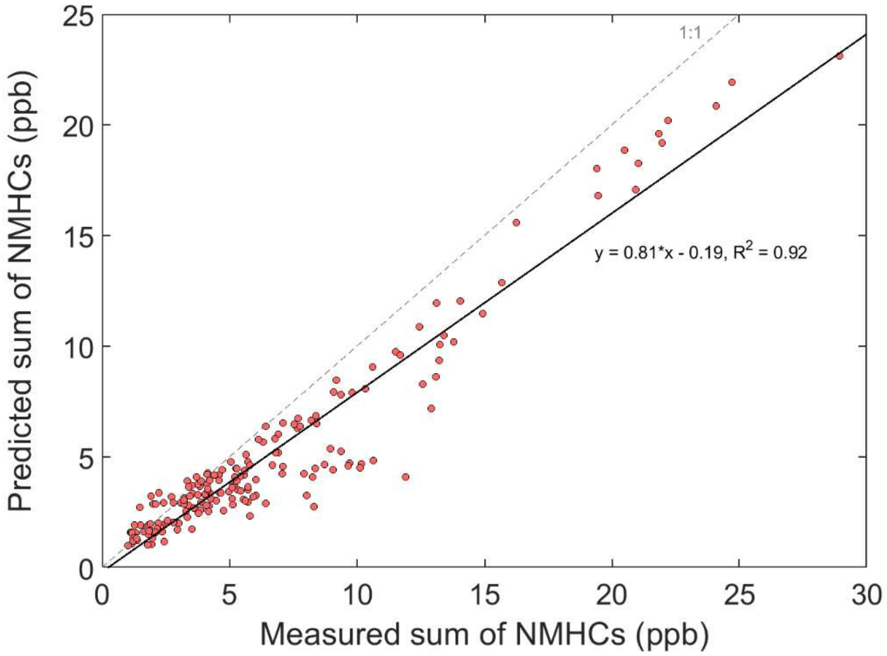

Supplementary Figure 18. Observed and predicted NMHC mixing ratios for the four factor solution. The solid line indicates the fitting and the dashed line the perfect 1:1 model.
